# Reading Notices

**Published:** 1910-12

**Authors:** 


### READING NOTICES

### A TRIUMPH IN PILL MAKING.

Parke, Davis & Co., confess that their soft-mass pill, which is now receiving so much favorable attention from the medical world, was for a long time a "hard nut" to crack. They had set out to produce by the soft-mass process a pill that should be a credit to their house and to manufacturing pharmacy. The task at first seemed simple enough. Here, as elsewhere, theory and practice were at variance. As a matter of fact, a good deal of experimentation had to be done. Time was consumed. Money was expended. In the end, of course, ingenuity triumphed.

In structure, the soft-mass pill, as manufactured by Parke, Davis & Co., consists of a plastic mass encompassed by a thin, soluble chocolate coating. It may be flattened between the thumb and finger like a piece of putty. An important advantage of the soft-mass pill is the readiness with which it disintegrates in the digestive tract. Another commendable feature is that, no heat being applied in the process, such volatile substances may be depended upon to contain just what the label says it contains.

Parke, Davis & Co., are putting out close to thirty formulas by the soft-mass process—all of them listed, we believe, in advertisements now appearing quite generally in the medical press. Practitioners under whose eyes these announcements do not happen to fall, may profitably write the company at its home offices in Detroit for a cpoy of a recently issued folder on "Soft-Mass Pills," which contains titles and complete formulas of all the pills now manufactured by Parke, Davis & Co., under the process referred to, together with some other important information.

---

### AN UNCONVENTIONAL COUGH SYRUP.

There are "cough syrups" without end. Some of them, it is needless to say, have little or no therapeutic value. Conversely, there are some that no physician need hesitate to prescribe. One of these—Syrup Cocillana Compound (P. D. & Co.)—is so exceptional in many particulars as to be worthy of special mention just now, when coughs are so plentifully in evidence. By its name no one would recognize it as a preparation for "coughs"

